# Atmospheric aerosol measurements from the ATSR-SLSTR series of dual-view satellite instruments 1995–2022

**DOI:** 10.1038/s41597-025-04694-6

**Published:** 2025-03-08

**Authors:** Kevin Pearson, Peter North, Andreas Heckel, Alberto Hornero, Stefan Kinne, Thomas Popp, Larisa Sogacheva, Jan Griesfeller

**Affiliations:** 1 Department of Geography, Swansea University, Swansea, UK; 2 FIELAX GmbH, Bremerhaven, Germany; 3 Instituto de Agricultura Sostenible, Spanish National Research Council, Córdoba, Spain; 4 Faculty of Engineering and Information Technology, The University of Melbourne, Melbourne, Australia; 5 Max Planck Institute for Meteorology, Hamburg, Germany; 6 German Aerospace Centre, Oberpfaffenhofen, Germany; 7 Finnish Meteorological Institute, Helsinki, Finland; 8 Norwegian Meteorological Institute, Postboks 43, Blindern, 0313 Oslo, Norway

**Keywords:** Atmospheric science, Scientific data

## Abstract

A data record, spanning 24 years, is presented of global atmospheric total aerosol optical depth and also the aerosol optical depth due to fine-mode constituents, typically of anthropogenic origin. Original measurements of reflectance were provided at approximately 1-km resolution by a series of dual-view satellite instruments: the Along-Track Scanning Radiometer 2 (ATSR-2), Advanced Along-Track Scanning Radiometer (AATSR), and Sea and Land Surface Temperature Radiometers (SLSTRs). These were processed to retrieve aerosol properties at 10-km resolution and then collated over daily and monthly timescales on a 1° × 1° latitude-longitude grid. Retrievals are evaluated against ground-based sun-photometer measurements from the Aerosol Robotic Network and Maritime Aerosol Network and compared to other satellite-derived datasets. The data record has implications for directly constraining the Earth’s radiation budget, allowing benchmarking and improvement of models to represent aerosol in the climate system, air quality monitoring and adding to the long-term record of emission trends related to sources such as fire, dust and sulphate pollution. After release, the SLSTR datasets will be regularly extended in time.

## Background & Summary

Aerosol optical depth (AOD) is an essential climate variable^1,2^. The interaction of aerosols within the climate system is complex and there is a large range in the estimated Radiative Forcing associated with aerosol effects of −2.0 to −0.6 W m^−2^ with 90% confidence^3^ over the period 1750 to 2014. Observations are required to contribute to reducing this uncertainty and to forming a climate data record for aerosols. In addition, global and regional aerosol measurements are needed for improved modelling of air quality and transport of pollution^4,5^. Ground-based sun-photometers capture the integrated column extinction, such as the mid-visible AOD, from attenuation measurements of the direct solar irradiance, in the absence of clouds, for solar spectral bands without trace-gas absorption. They are, however, limited in their geographical distribution. Satellite-data-based AOD retrievals, in contrast, offer near-global coverage. They are less certain because AOD must be deduced using indirect inversion techniques based on radiance measurements at the top of the atmosphere (TOA) when viewing the Earth’s heterogeneous surface. They are thus subject to assumptions with regard to surface properties and aerosol composition. Uncertainties in retrievals can be reduced in a number of ways: improved radiometric performance and calibration of satellite instruments; use of more sophisticated techniques such as using multi-angular and multi-spectral information to deduce the underlying surface reflectances more accurately; and better representation of the optical properties of different aerosol types and mixtures present in the retrieval.

This paper presents a data record of aerosol derived from a family of dual-view satellite instruments: the Along-Track Scanning Radiometers (ATSR-2 and AATSR, collectively (A)ATSR), and the Sea and Land Surface Temperature Radiometers (SLSTR-A and -B, onboard Sentinel-3A and -3B, respectively). These cover the period from the start of the European Remote-Sensing Satellite 2 (ERS-2) mission to the end of the Environmental Satellite (Envisat) mission (1995–2012) and from the start of the Sentinel-3A mission to the present day (2016–2022). These instruments were designed to make highly-stable measurements of the Earth’s surface at several visible and near-infrared wavelengths, using both a nadir and an inclined viewing angle, in order to derive sea surface temperature with very high accuracy; however, they are also successfully utilized to retrieve atmospheric aerosols. ATSR-2 and AATSR were near-identical instruments and utilised a nadir and forward view that covered the same swath on the Earth’s surface. The predecessor instrument ATSR-1 is not included as it lacked the three visible channels required by the retrieval method. The two SLSTR instruments are identical and similar to the ATSRs but use a nadir and rearward-facing view (termed “oblique”). This oblique view does not cover the full swath-width of the nadir view which is wider (1400 km) than that of the ATSRs. Here we present a common retrieval algorithm for the instrument series, and validate the global record of aerosol optical depth and its fine mode component.

**Fig. 1:**
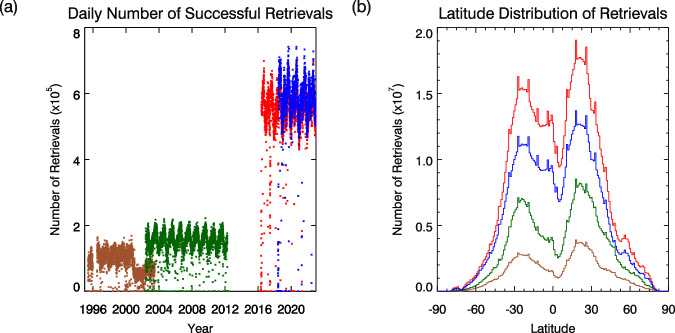
Comparison of retrievals by ATSR-2 (brown), AATSR (green), SLSTR-A (red) and SLSTR-B (blue): (**a**) number of successful daily retrievals; (**b**) total number of retrievals for each 1° latitude band.

The retrieval algorithm operates on level 1B (L1B) data from the instruments binned to 9 × 9 super-pixels and generates level 2 (L2) output using the same grid and format as the input data. These are then converted to a common level 2P netCDF format file for each orbit that uses a 4008 × 2004 sinusoidal grid with 10 km resolution. The number of successful retrievals per day and their latitude distribution are shown in Fig. 1. The mean number of contributing retrievals per day increases over the course of the record from 1 × 10^5^ to 6 × 10^5^ for a single instrument. The datasets are provided as collated level 3 (L3C) products on 1° × 1° latitude-longitude grids in two forms, being composited over either daily or monthly periods. Each AOD value is accompanied by uncertainty information propagated from L2. For SLSTR, the mean time to complete all the steps necessary to process an orbit of compressed L1B data to L2 was 26 min. Generating the L3C daily and monthly data from the L2 files took an additional 100 min per month.

## Methods

The aim of the retrieval algorithm is to provide AOD and associated aerosol properties, globally, over all ocean and land surfaces, which are free of cloud and ice, from the (A)ATSR-SLSTR satellite instrument series. A key challenge is the separation of the contribution from atmospheric scattering from that of the surface, which is often higher in magnitude. The origin of the current algorithm is a model inversion framework^6^ based on simultaneous retrieval of surface reflectances and AOD, using a dual-angle retrieval first demonstrated for ATSR-2 over land regions. This was later extended over ocean using a simple dark surface model at which point the first global validation was performed^7^. Subsequently, the algorithm was developed through a series of iterations of innovation and evaluation under the European Space Agency’s Climate Change Initiative aerosol project^8^ (Aerosol CCI), and extended to the AATSR and SLSTR instruments. Within this framework, the algorithm has been developed to allow retrieval of both aerosol model and size, parameterised by the ratio of fine to coarse mode aerosols, and includes a model of scattering by non-spherical dust particles. The ocean model was developed to include representation of sun and sky glint, absorption by ocean pigments and surface foam. Over land, a number of innovations have been introduced to improve stability and to model both the spectral and angular distribution of the surface reflectance. In particular, the latest algorithm includes a new cost-function term relating the red and mid-infrared ((A)ATSR) or shortwave-infrared (SLSTR) reflectances over land, improved calibration for the SLSTR measurements and several bug fixes. These significantly improve performance compared to the previous (A)ATSR (v4.33) and SLSTR (v.1.12) versions^9^, particularly for SLSTR and over bright surfaces. The final algorithm^10,11^ has been applied to the full global record from the four dual-view satellite instruments and validated to produce the dataset presented here.

### Input data

Input data for (A)ATSR retrievals are taken from the v2.1/v3.0 L1B archive^12,13^ and the algorithm uses the channels in band 1 (550 nm), 2 (670 nm), 3 (865 nm) and 4 (1610 nm). The SLSTR retrieval utilises the latest data version available (v3 or v4) at the time of processing in the Centre for Environmental Data Analysis archive^14,15^. This algorithm uses the observations in channels S1 (550 nm), S2 (670 nm), S3 (868 nm), S5 (1613 nm) and S6 (2256 nm) with the correction factors applied recommended by in-flight calibration studies^16^. Meteorological data are included in the input L1B datasets. Of these, currently only the surface atmospheric pressure is used by the retrieval. The periods covered and data volumes involved are given in Table 1. The L1B (A)ATSR data are provided per orbit, while the SLSTR data are generated as granules lasting approximately 3 minutes. The SLSTR data can thus be pre-screened to process only those scenes that occur on the sunlit (descending) part of the orbit. The geolocated and calibrated data pixels, which are provided on a swath-based grid, are screened for cloud and spatially aggregated to form a 9 × 9 super-pixel, intended to minimise any impact of co-location errors between the two instrument views. These super-pixels are processed by the retrieval algorithm and the returned quantities are output as L2 products on the same grid.

**Table 1:** Summary of the input data sensors, periods and volumes used and generated.

Sensor	Date Range Used (Year/Month/Day)	L1 data volume (approx.) (TB)	L3C data volume generated (GB)
ATSR-2	1995/06/01-1995/12/22, 1996/07/01-2003/06/22	25	3.1
AATSR	2002/05/20-2012/04/08	41	5.3
SLSTR-A	2016/05/01-2022/12/31	475	6.4
SLSTR-B	2018/05/09-2022/12/31	330	4.5

The ATSR-2 sensor suffered an anomaly from 1995/12/22-1996/06/30 during which time no data is available.

### Cloud detection

The retrieval scheme requires input pixels to be free of cloud, snow, ice and sun glint. These are identified during a pre-processing stage that applies tests separately to the two views. Pixels flagged as snow, ice or glint in the L1B input products are immediately excluded. A cloud-mask is then applied based on thresholds for TOA channel reflectances and channel combinations. The surrounding 8 pixels to any pixel flagged as cloudy are also excluded. Over ocean, an additional test for high levels of glint is applied that uses the modelled bi-directional reflectance distribution function (BRF) values in the LUT appropriate to the viewing geometry of the pixel. If this modelled BRF exceeds 0.008 for the 1610 nm channel, the pixel is excluded from further processing.

### Retrieval

The retrieval process optimises the values of fine-mode fraction (FMF) and AOD consistent with the observations. The components within the fine and coarse modes are split between strongly absorbing/weakly absorbing and dust/sea-salt respectively^17^. While FMF is a retrieved quantity, the divisions between these fine and coarse sub-types are taken from a monthly 1° × 1° climatology^18^.

The retrieval scheme operates by minimising the difference between the surface reflectances (${R}_{{\rm{surf}}}$) inferred from TOA observed reflectances and those provided by an *a priori* model (${R}_{\mathrm{mod}}$). The models differ for land or ocean surfaces, determined by the L1B data flags for the super-pixel, with the land model being significantly more complex due to the more heterogeneous nature of the land surface. The radiative transfer calculation to derive the intensity of light arriving at TOA through a known atmospheric profile is, conceptually, a straight-forward problem. The properties of the atmosphere at each level determine the amount of absorption and scattering out of the line of sight. In detail, however, it can be a more complex and slower computational process. For reasons of speed, the process is represented by a set of parameters stored in a look-up table (LUT) that are pre-computed by detailed modelling using the 6SV code^19–21^.

**Fig. 2:**
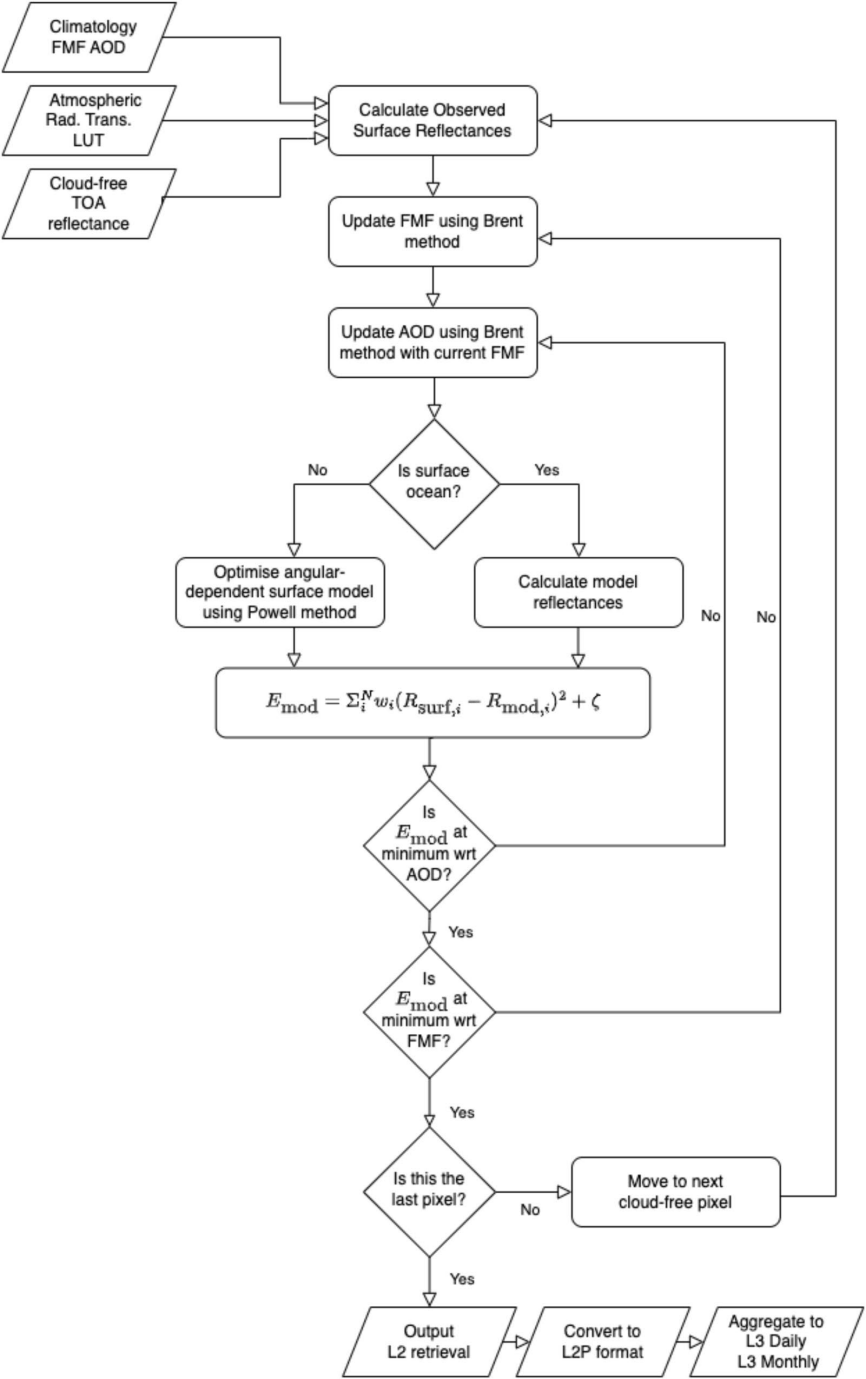
Overview of the algorithm and processing of the retrieval system.

The retrieval proceeds in two steps. First, the LUT is used to derive an “observed” surface reflectance for each channel and each view for an initial atmospheric profile and composition. Next, a nested iterative process minimises the (land or ocean) surface model reflectances against these surface reflectances using FMF and AOD at 550 nm (AOD_550_) as the free parameters. Each channel is weighted using appropriate error estimates for the observed and modelled reflectances and the cost function includes extra additive terms that ensure numerical stability and prevent non-physical results. The processing scheme is illustrated in Fig. 2 and described in more detail below. Full details of the algorithm are given in the respective Algorithm Theoretical Basis Documents^10,11^.

#### Radiative transfer

As a first step, the satellite observations at TOA in each band are expressed as reflectances (${R}_{{TOA}}$). This is simply the ratio between the fluxes due to the observed (${L}_{{TOA}}$) and solar (${F}_{0}$) radiances. Any adjustment factors due to updates in channel calibration are applied at this stage. Thus,

$${R}_{{TOA}}=\frac{\pi {L}_{{TOA}}}{{F}_{0}\cos ({\theta }_{s})}$$

where ${\theta }_{s}$ is the solar zenith angle. This TOA reflectance can be related^7,19^ to the surface directional reflectance (${R}_{{\rm{surf}}}$) by

$${R}_{{TOA}}\left({\theta }_{v},{\theta }_{s},\phi \right)={R}_{{atm}}\left({\theta }_{v},{\theta }_{s},\phi \right)+T\left({\theta }_{s}\right)T\left({\theta }_{v}\right)\frac{{R}_{{surf}}\left({\theta }_{v},{\theta }_{s},\phi \right)}{1-{\rho }_{{atm}}{R}_{{surf}}^{{\prime} }}$$

Here ${\theta }_{v}$ is the satellite viewing zenith angle, *ϕ* is the sun to satellite relative azimuth angle, ${R}_{{atm}}$ is the contribution of atmospheric reflectance to the total, T is the atmospheric transmission for a given zenith angle and ${\rho }_{{atm}}$ is the atmospheric bi-hemispherical albedo. The ${R}_{{surf}}^{{\prime} }$ term is the surface reflectance for multiple scatterings of light. With the simplifying assumption that ${R}_{{surf}}^{{\prime} }\approx {R}_{{surf}}$,this equation allows ${R}_{{\rm{surf}}}$ to be determined for a given ${R}_{{TOA}}$ if ${\rho }_{{atm}}$, ${R}_{{atm}}$ and T are known. These latter quantities are pre-computed and stored in a LUT for each observation band and aerosol mixture over a range of viewing angles and AODs as described in the auxiliary data section below.

#### Ocean surface model

For retrievals over ocean, the surface model follows the established method^22^ of calculating the surface BRF (${R}_{{\rm{ocean}}}$) as a sum of contributions from whitecaps, glint and open water

$${R}_{{\rm{ocean}}}={R}_{{wc}}+(1-{f}_{w}){R}_{{gl}}+(1-{R}_{{wc}}){R}_{{ow}}$$

The terms are taken separately from existing models for glint^23^, foam and spectral reflectance^22,24^ and case I water reflectance with pigment concentration dependence^25^. Fixed input values are used for wind speed (3 m s^−1^) and pigment concentration (0.1 mg m^−3^). The calculation is coupled with the 6S radiative transfer model to account for sky glint. The total BRF is pre-computed for a range of conditions and stored in the LUT described in the auxiliary data section below.

Retrievals are carried out over the full-swath for locations wherever at least one view is clear, but make use of the observations from both views if they are available. Observations in Band1/Channel S1 (550 nm) are excluded due to their sensitivity to chlorophyll and sediment.

#### Land surface model

The heterogeneity of the land surface compared to the ocean requires a more sophisticated approach. The method employed here builds on that of a previous version^26^ and simultaneously estimates FMF, AOD_550_ and surface reflectances by applying a constraint on the angular variation of the surface reflectances. As such, it requires both nadir and inclined observations to be available for the retrieval to proceed. Such an approach has the advantage of requiring no *a priori* information about the surface. A physical model of spectral change with view angle^26^ leads to an expression for the modelled bi-directional reflectance

$${R}_{{\rm{land}}}\left(\lambda ,\Omega \right)=\left(1-D\left(\lambda \right)\right)\nu \left(\Omega \right)\omega \left(\lambda \right)+\frac{\gamma \omega (\lambda )}{1-g}\left[D\left(\lambda \right)+g\left(1-D\left(\lambda \right)\right)\right]$$

where *γ* is the fraction contributing to higher-order scattering (fixed at 0.35), g = 1 − *γω*(*λ*), D is the fraction of diffuse light, *λ* is the wavelength and Ω indicates either of the two viewing geometries (i.e. nadir or inclined view). The free parameters in the retrieval are the structural *ν*(Ω) and spectral *ω*(*λ*). Stability considerations lead to fixing ν = 0.5 for the nadir view. Including FMF and AOD_550_, this leaves a total of 8 free parameters to be determined from the 10 observations for SLSTR and 7 free parameters from 8 observations for (A)ATSR. This model fits the surface behaviour well for a wide variety of surface types and has the benefit that it differs from that of atmospheric scattering^26^. The inversion is thus able to discriminate well between these two contributions to the TOA observations.

The reliance on a multi-angular constraint when over land means that for SLSTR, retrievals can occur only for the narrower dual-view region of the swath, rather than for the full swath-width when over ocean surfaces.

#### Inversion

FMF and AOD_550_ are retrieved by nested minimisations of a weighted least squares cost function of the difference between the deduced “observational” surface reflectances and the modelled reflectances. The weights for each channel are provided by the uncertainties for modelled and observed reflectances added in quadrature so that the cost-function represents a χ^2^ goodness of fit measure of the reflectances.

Additional terms are added to the cost function as regularising constraints. To improve performance over difficult, and particularly, bright surfaces, a cost is added for land surfaces based on the difference between the modelled reflectances at long and short wavelengths. This follows similar methods to the dark target approach used for MODIS^27^, MERIS/AATSR synergy and SLSTR/OLCI synergy^28^ products. In the absence of a blue channel, this formulation encourages a correlation between the reflectances at 670 nm and either 1610 nm (for (A)ATSR) or 2256 nm (for SLSTR). The coefficients for the relative weight and proportionality of this correlation have been optimised for low- and high-NDVI cases and are interpolated to the current NDVI within the surface model optimisation.

Other terms are added to the cost function to ensure numerical stability, prevent unphysical retrievals (e.g. negative reflectances) and to discourage extreme variations from climatological values.

### Auxiliary data

The optical properties of atmospheric aerosols are represented as a mixture of 2 sub-types each of “fine-mode” or “coarse-mode” components. The fine-mode fraction gives the split between fine-mode AOD and coarse-mode AOD. The fine mode consists of “weak absorbing” or “strong absorbing” sub-types while the coarse mode consists of dust and sea salt. The fundamental optical properties and parameters for an assumed log-normal size distribution of these components are those defined for Aerosol CCI^17^ and are listed in Table 2. This information was used to calculate the scattering and wavelength dependent optical properties of the sub-types; using a Mie code for the spherical particles and a T-matrix code for dust^29,30^. The 35 mixtures that covers all possible combinations of the four sub-types in 25% steps of the total are used to generate LUTs that represent the radiative transfer process.

**Table 2:** Summary of the physical and optical properties at 550 nm of the four components considered in the radiative transfer calculation.

Aerosol Component	Refractive Index (Real)	Refractive Index (Imaginary)	Effective Radius (μm)	Radius Geometric Std. Dev.	Radius Std. Dev.	Median Radius
Dust	1.56	0.0018	1.94	1.822	0.6	0.788
Sea Salt	1.4	0	1.94	1.822	0.6	0.788
Fine-mode (weakly absorbing)	1.4	0.003	0.140	1.7	0.53	0.07
Fine-mode (strongly absorbing)	1.5	0.040	0.140	1.7	0.53	0.07

The sizes of aerosol particles are assumed to follow a log-normal distribution.

The retrieval uses monthly climatological values based on a multi-model ensemble median^18^ for the proportions of the sub-components within the fine or coarse modes. These are provided by a LUT on a 1° by 1° latitude-longitude grid. The algorithm does, however, retrieve the fine-mode to coarse-mode mixing ratio. Subsequent calculations find values for the variables appropriate to this mixture by using tetrahedral interpolation between the 35 model mixtures.

For reasons of speed, radiative transfer in the retrieval process is approximated using tabulated values for the variables ${R}_{{atm}}$, *T*, ${\rho }_{{atm}}$ and *D* that are stored in a LUT. These are derived from the 6S radiative transfer code^19–21^. The ocean surface reflectance ${R}_{{\rm{ocean}}}$ is stored similarly in a separate LUT. At run time, these are interpolated in a piecewise linear way along their physical dimensions which are listed in Table 3.

**Table 3:** Dimensional dependencies of variables used in the retrieval that are interpolated from LUTs.

LUT Dimension		Variable				
Name	Symbol	*R*_*atm*_	*T*	*ρ*_*atm*_	*D*	*R*_*ocean*_
Model	*M*	X	X	X	X	X
Band	λ	X	X	X	X	X
Surface Pressure	*Ps*	X	X	X	X	
AOD 550 nm	τ_550_	X	X	X	X	X
Viewing Zenith Angle	θ_*v*_	X				X
Sun Zenith Angle	θ_*s*_	X	X		X	X
Relative Azimuth	ϕ	X				X

### Uncertainty propagation

Uncertainties are derived from the shape of the cost function. For a correctly normalised χ^2^, the uncertainty is given by the square root of its curvature in the region of the minimum value. However, the correct weighting for the model and observed terms are difficult to determine *a priori*. Additionally, the regularising constraints and, in particular, the spectral correlation term for land surfaces, causes the cost function to deviate from this ideal. The uncertainty values for the retrieved AOD_550_ are thus assumed to remain proportional to the curvature but decreased by a single scale factor common to either the two (A)ATSR or two SLSTR instruments.

### Level 2 output data

AOD_550_ and FMF are the two directly retrieved quantities from the above algorithm. A post-retrieval filtering step is applied to the AOD_550_ field to remove super-pixels potentially affected by undetected cloud^31^. To pass this step, there must be at least 3 other successful retrievals in the neighbouring 8 super-pixels, and the standard deviation of the AOD in the 9 super-pixels must be less than 0.15 or 80% of the mean AOD, whichever is smaller. AOD_550_ for each super-pixel is included in the output while the FMF is included in the form of the fine-mode aerosol optical depth (being the product of the two quantities). Each retrieval is carried out with a fixed mixing fraction for the sub-types within the fine and coarse modes that is provided by the local, monthly climatology. Thus, the assumed optical properties of the aerosols are also fixed for a given retrieval. These same assumptions are used, in combination with the retrieved AOD_550_ and FMF, to derive self-consistently additional quantities that are also included in the output. It should be noted that these values are, as a result, dependent on the choices that have been made with regard to the pre-defined aerosol components.

#### Spectral AOD

The ratio ${r}_{550,\lambda }$ of the AOD at a particular instrument wavelength (*λ*) to the AOD_550_ is generated with, and included alongside, the quantities in the atmospheric LUT for the 35 mixtures of the 4 component sub-types. The appropriate values of this ratio for each retrieval are derived from interpolation between these mixtures based on the retrieved FMF and combined with the retrieved AOD_550_ to provide the AOD at wavelengths 670, 870 and 1610 nm.

#### Ångstrøm exponent

The wavelength dependence of AOD is often approximated as a power-law with an Ångstrøm exponent, α. Here it is generated using the above ${r}_{\mathrm{550,865}}$ value that has been interpolated to the retrieved FMF from the values stored in the atmospheric LUT. Thus,

$$\alpha =\frac{\mathrm{ln}\left({r}_{550,865}\right)}{\mathrm{ln}\left(550\right)-\mathrm{ln}(865)}$$

#### Single-scattering albedo

Values for the single-scattering albedo (SSA) are calculated during the 6S radiative transfer modelling and included with the other contents of the atmospheric LUT for 35 mixtures at each wavelength. In common with the ratio ${r}_{550,\lambda }$, these are interpolated according to the retrieved FMF and the SSA at 550 nm is included in the output.

#### Absorbing AOD

The AOD due to absorption (${\tau }_{{abs}}$) is directly calculated from the SSA (${{\rho }}_{{ss}}$) and AOD_550_ (${\tau }_{550}$) and included in the output. It is given by

$${\tau }_{{abs}}=\left(1-{{\rho }}_{{ss}}\right){\tau }_{550}$$

#### Dust AOD

The contribution of dust to the AOD (${\tau }_{{dust}}$) is derived from a combination of the retrieved FMF (*f*), AOD_550_ and the fraction of dust (${f}_{{dust}}$) in the coarse mode that was provided by the climatology LUT.

$${\tau }_{{dust}}=\left(1-f\right){f}_{{dust}}{\tau }_{550}$$

### Level 3 Gridded products

**Fig. 3:**
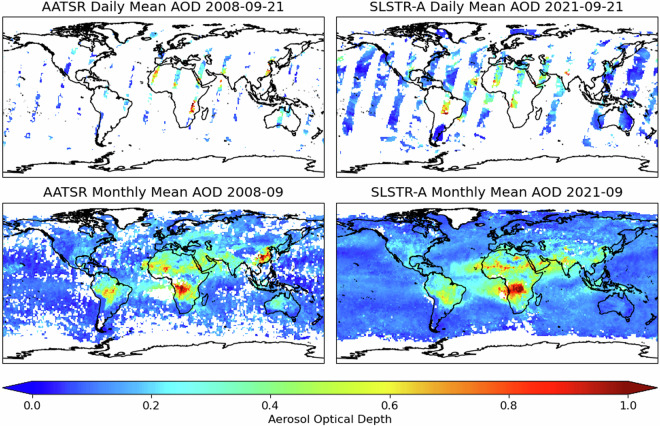
Example daily and monthly mean AOD at 550 nm fields for AATSR and SLSTR-A. The wider swath of the SLSTR instruments results in significantly improved global coverage.

**Fig. 4:**
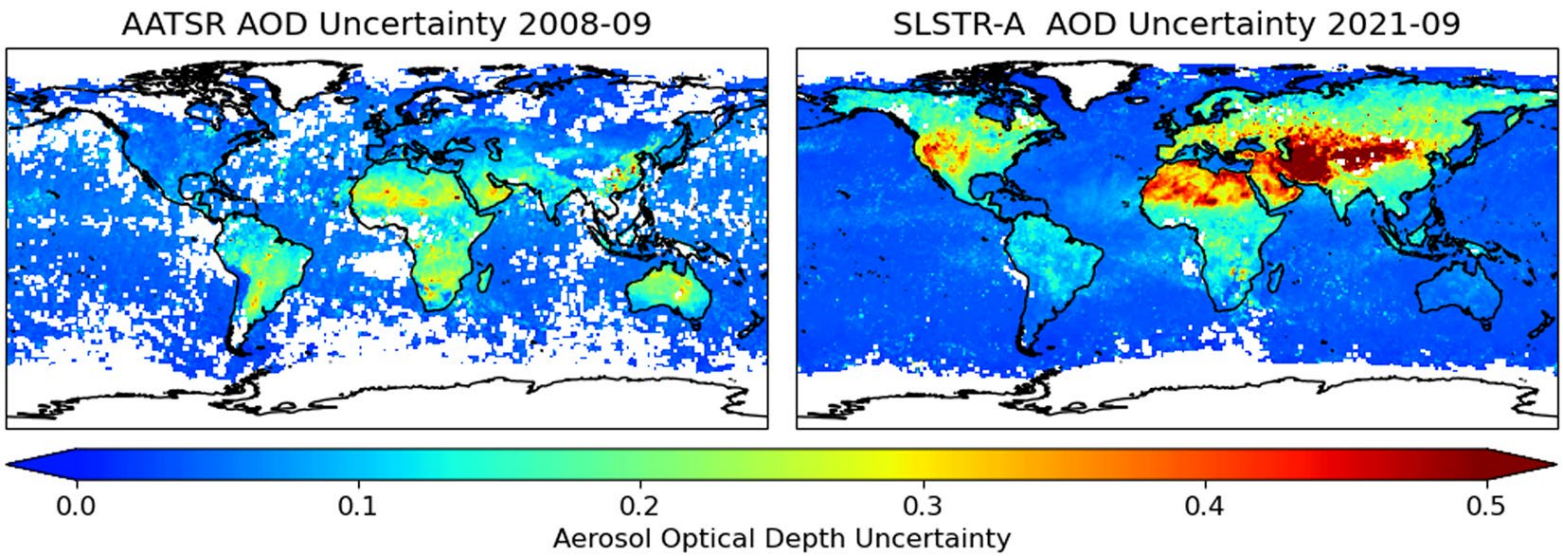
Example uncertainty fields for the monthly mean AOD at 550 nm for AATSR and SLSTR-A. The reversal of the geometry of the forward and inclined views results in reversal of the hemisphere with greatest uncertainties for retrievals over land.

The dataset is comprised of L3C products formed from the L2 data aggregated on a 1° × 1° latitude-longitude grid and accumulated over either daily or monthly timescales. The mean and standard deviation are included for all quantities. For AOD, both propagated uncertainties and statistical information relating to the uncertainties of the super-pixels contributing to the cell are included. Examples of daily and monthly mean AOD fields are shown in Fig. 3. The greater coverage provided by the SLSTR swath compared to AATSR is readily apparent in the daily data. This results in a more frequent revisit time for each of the SLSTR instruments and improved sampling in the monthly data. The data gaps apparent in the monthly AATSR data are expected to occur preferentially in persistently cloudy areas. Since these areas will tend to have lower AOD, there is the potential to introduce a bias in the global AOD to be low relative to the SLSTR data. Corresponding AOD uncertainty fields for the monthly data are shown in Fig. 4. The reversal of the orientation of the forward to oblique views results in a reversal of which hemisphere views solar irradiance being forward- or back-scattered. The weaker back-scattered atmospheric signal, with corresponding increase in the land signal, leads to greater uncertainty in the retrieved quantities. While the patterns are reliable, the absolute values probably overestimate the true uncertainties. Pessimistic assumptions regarding the correlation of the contributing uncertainties have been employed, while the true spatio-temporal correlation scales remain areas of research. Similarly, all the retrieval uncertainty has been ascribed to AOD rather than shared with the FMF.

## Data Records

The data record^32^ can be retrieved from the Centre for Data Analysis archive (https://archive.ceda.ac.uk/).

Daily and monthly files contain identical variables for a given sensor. The (A)ATSR files include the same variables as SLSTR-A and -B sensors and additionally fitted surface reflectance, cloud fraction and surface type information. The contents are listed in Tables 4, 5. Summary information for the datasets is given in Tables 6, 7, including the names, data volumes and digital object identifiers. The data are released under the Creative Commons Attribution 4.0 International License (CC BY 4.0, http://creativecommons.org/licenses/by/4.0/).

**Table 4:** Names and descriptions of variables in the dataset files.

Variable Name	Long Name
**Dimensions:**	
latitude	Latitude
longitude	Longitude
**Retrieved:**	
pixel_count	number of retrieved pixels in grid cell
AOD550_mean	aerosol optical thickness at 550 nm
AOD550_sdev	standard deviation aerosol optical thickness at 550 nm
FM_AOD550_mean	fine mode AOD
FM_AOD550_sdev	standard deviation of fine mode AOD
AOD550_uncertainty	propagated L2 uncertainty in aerosol optical thickness at 550 nm
AOD550_uncertainty_mean	mean of L2 uncertainty on AOT at 550 nm
AOD550_uncertainty_min	minimum L2 uncertainty on AOT at 550 nm
AOD550_uncertainty_max	maximum L2 uncertainty on AOT at 550 nm
AOD550_uncertainty_sdev	standard deviation of L2 Uncertainty on AOT at 550 nm
**Derived:**	
AOD < band > _mean	aerosol optical thickness at < band > nm
AOD < band > _sdev	standard deviation aerosol optical thickness at < band > nm
AOD < band > _uncertainty	propagated L2 uncertainty in aerosol optical thickness at < band > nm
AOD < band > _uncertainty_mean	mean of L2 uncertainty on AOT at < band > nm
AOD < band > _uncertainty_min	minimum L2 uncertainty on AOT at < band > nm
AOD < band > _uncertainty_max	maximum L2 uncertainty on AOT at < band > nm
AOD < band > _uncertainty_sdev	standard deviation of L2 Uncertainty on AOT at < band > nm
ANG550_870_mean	angstrom exponent computed on AOD550nm and AOD870nm
ANG550_870_sdev	standard deviation angstrom exponent computed on AOD550nm and AOD870nm
D_AOD550_mean	non-spherical dust AOD
D_AOD550_sdev	standard deviation non-spherical dust AOD
AAOD550_mean	absorbing AOD
AAOD550_sdev	standard deviation absorbing AOD
SSA550_mean	single scattering albedo at 550nm
SSA550_sdev	standard deviation of SSA

The marker < band > can be replaced by “550”, “670”, “870” or “1600” for the values at the indicated wavelength.

**Table 5:** Names and descriptions of additional variables included the ATSR-2 and AATSR dataset files only.

Variable Name	Long Name
surface_reflectance < band > _mean	mean bidirectional surface reflectance (nadir)
surface_reflectance < band > _sdev	standard deviation mean bidirectional surface reflectance (nadir)
cloud_fraction_mean	mean fraction of cloud flagged pixels in 10 km bin
cloud_fraction_sdev	standard deviation mean fraction of cloud flagged pixels in 10 km bin
surface_type_number_mean	mean land fraction
surface_type_number_sdev	standard deviation mean land fraction

The marker < band > can be replaced by “550”, “670”, “870” or “1600” for the values at the indicated wavelength.

**Table 6:** Data record information for gridded collated level 3 (L3C) daily Swansea University aerosol products.

Dataset title	SU Daily Aerosol (A)ATSR L3C v4.35.1	SU Daily Aerosol SLSTR L3C v1.14.1
Full name	Swansea University Aerosol Algorithm: (Advanced) Along-track Scanning Radiometers Daily Collated Level-3 Product v4.35.1	Swansea University Aerosol Algorithm: Sea and Land Surface Temperature Radiometers A and B Daily Collated Level-3 product v1.14.1
Basic description	Aerosol optical depth and fine-mode fraction from (Advanced) Along-track Scanning Radiometers, monthly collation on a 1° × 1° latitude-longitude grid, 1995–2012	Aerosol optical depth and fine-mode fraction from Sea and Land Surface Temperature Radiometers, monthly collation on a 1° × 1° latitude-longitude grid, 2016–2022
Total data volume	7.0GB	10.2GB
Digital Object Identifier	10.5285/397b2da3a0d04bde8e5e1e341c829422	10.5285/f18f81e6fe014e5ab7b847f282f9de7b

**Table 7:** Data record information for gridded collated (level 3C) monthly Swansea University aerosol products.

Dataset title	SU Monthly Aerosol (A)ATSR L3C v4.35.1	SU Monthly Aerosol SLSTR L3C v1.14.1
Full name	Swansea University Aerosol Algorithm: (Advanced) Along-track Scanning Radiometers Monthly Collated Level-3 Product v4.35.1	Swansea University Aerosol Algorithm: Sea and Land Surface Temperature Radiometers A and B Monthly Collated Level-3 Product v1.14.1
Basic description	Aerosol optical depth and fine-mode fraction from (Advanced) Along-track Scanning Radiometers, monthly collation on a 1°×1° latitude-longitude grid, 1995–2012	Aerosol optical depth and fine-mode fraction from Sea and Land Surface Temperature Radiometers, monthly collation on a 1°×1° latitude-longitude grid, 2016–2022
Total data volume	1.3GB	0.7GB
Digital Object Identifier	10.5285/f677ad3b44c24d5e8701153f14ab39e4	10.5285/a89007aa668d4e2f940dbb3d3dfcc3dc

## Technical Validation

**Fig. 5:**
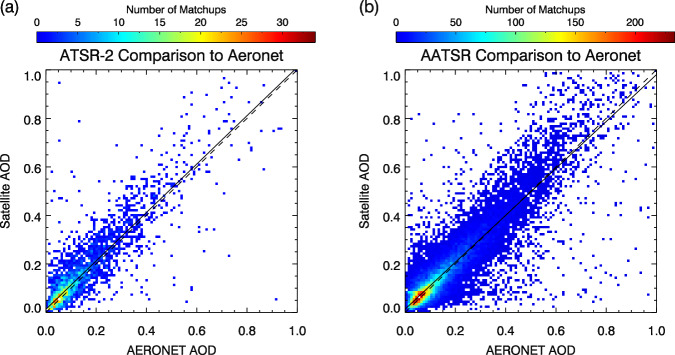
Comparison of ATSR AOD retrievals to AERONET stations: (**a**) ATSR-2; (**b**) AATSR. A weighted straight-line fit is shown for both instruments (solid line) along with a 1:1 line (dashed).

**Fig. 6:**
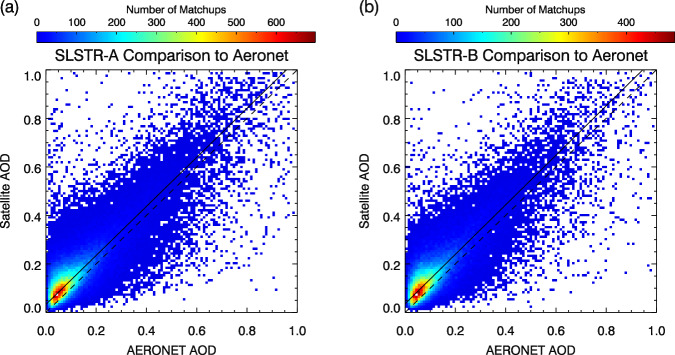
Comparison of SLSTR AOD retrievals to AERONET stations: (**a**) SLSTR-A; (**b**) SLSTR-B. A weighted straight-line fit is shown for both instruments (solid line) along with a 1:1 line (dashed).

**Fig. 7:**
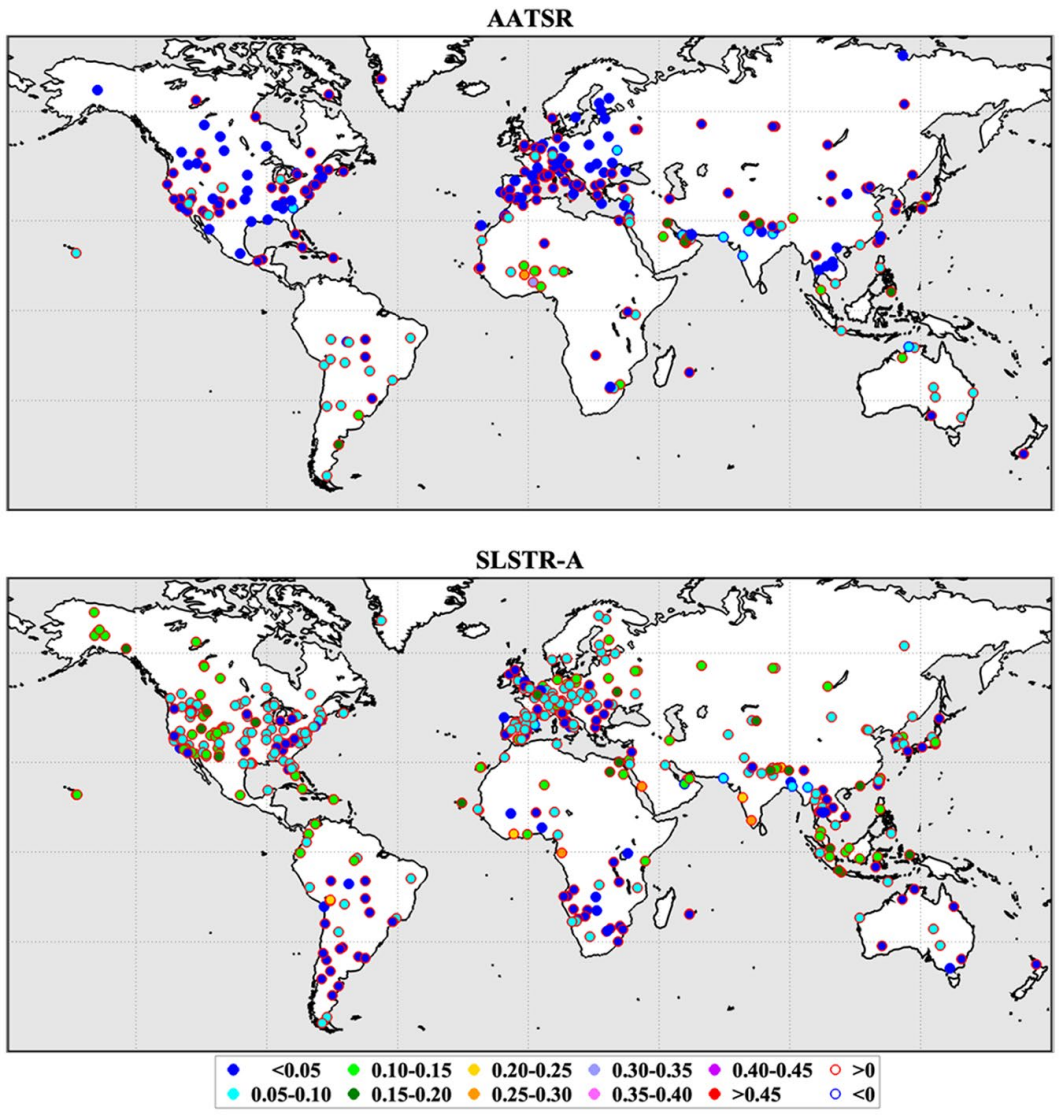
Global distribution of the mean offset between satellite and AERONET AOD for AERONET locations with at least 10 matches for AATSR and SLSTR-A.

Retrieved values of AOD at L2 were compared to co-incident measurements made by ground-based Aerosol Robotic Network (AERONET) stations^33,34^ to validate the retrieval scheme. Ground station and satellite measurements within 45 minutes and 15 pixels of a satellite-ground station over-pass were included in a matchup and a mean and standard deviation for the AOD at 550 nm was calculated for both sources. These are shown in Fig. 5 for ATSR-2 and AATSR and in Fig. 6 for SLSTR-A and SLSTR-B. All matchups found within the datasets’ full time range are included. Summary statistics are given in Table 8. These include the fraction that fall within the breakthrough measurement requirement envelope for the difference between the two; being the maximum of 0.03 or 10% defined by the Global Climate Observing System^1^ (GCOS). Also listed are the fraction meeting the same requirement after adjusting each measurement for the overall dataset bias relative to AERONET. Figure 7 illustrates the geographical variation in the differences between the satellite and AERONET AODs for station locations with at least 10 successful matches. Most clearly, the spatial distributions of these biases reflect the swapping of the hemisphere with greater uncertainty, following the swapping of the orientation of the instruments’ inclined views. Also apparent is the relative over-representation of AERONET stations in the Northern Hemisphere. This will result in different systematic effects on the global matchup statistics for the two pairs of instruments.

**Table 8:** Summary statistics for matchups of satellite measurements of AOD to AERONET station measurements.

Sensor	Mean AOD	Bias	RMSE	Correlation	GCOS fraction	GCOS_b fraction	Matches
ATSR-2	0.171	0.023	0.115	0.819	48.6	47.0	2,812
AATSR	0.175	0.011	0.094	0.864	53.4	54.1	24,554
SLSTR-A	0.140	0.044	0.097	0.832	34.3	42.4	88,904
SLSTR-B	0.138	0.049	0.099	0.827	32.2	41.4	62,551

The mean value of the AERONET station AOD across all matches is given, along with the relative bias and root-mean-square difference of the satellite values.

**Fig. 8:**
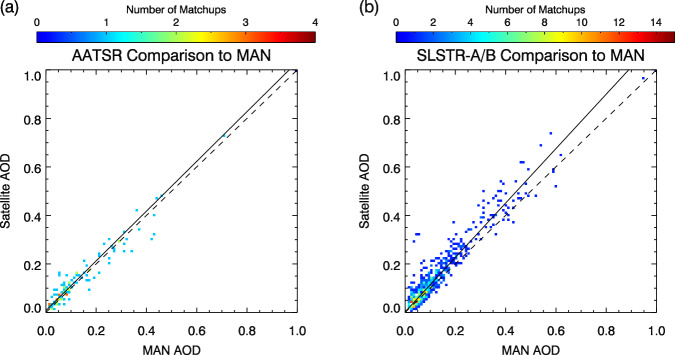
Comparison of satellite AOD retrievals with Maritime Aerosol Network measurements: (**a**) ATSR-2 and AATSR; (**b**) SLSTR-A and SLSTR-B. A weighted straight-line fit is shown for both instruments (solid line) along with a 1:1 line (dashed).

A similar comparison was carried out for ship-based measurements from the Maritime Aerosol Network (MAN)^35^. The record for these sources begins in 2004, after the end of the ATSR-2 mission and there are fewer measurements than for AERONET ground stations. Data are thus presented for AATSR and then for SLSTR-A and SLSTR-B combined. These are shown in Fig. 8 and summarised in Table 9.

**Table 9:** Summary statistics for matchups of satellite observations to Marine Aerosol Network measurements.

Sensor	Mean AOD	Bias	RMSE	Correlation	GCOS fraction	GCOS_b fraction	Matches
AATSR	0.136	0.006	0.036	0.960	72.7	73.6	110
SLSTR-A/SLSTR-B	0.123	0.028	0.049	0.955	62.7	63.5	611

Retrieved values for the *fine-mode* AOD were compared to values in the AERONET SDA product. The AERONET values were adjusted from a reference wavelength of 500 nm to 550 nm using the accompanying Ångstrøm exponent and the logarithmic spectral derivative of the Ångstrøm exponent. The matchup criteria were identical to the previous overall AOD comparisons and summary statistics are given in Table 10.

**Table 10:** Summary statistics for matchups of satellite measurements of fine-mode AOD to AERONET station measurements.

Sensor	Mean AOD	Bias	RMSE	Correlation	GCOS fraction	GCOS_b fraction	Matches
ATSR-2	0.107	0.037	0.100	0.791	43.2	47.2	1,929
AATSR	0.103	0.028	0.078	0.841	51.5	57.2	18.504
SLSTR-A	0.088	0.034	0.101	0.700	40.1	40.3	83,376
SLSTR-B	0.087	0.040	0.105	0.687	37.6	37.4	69,022

**Fig. 9:**
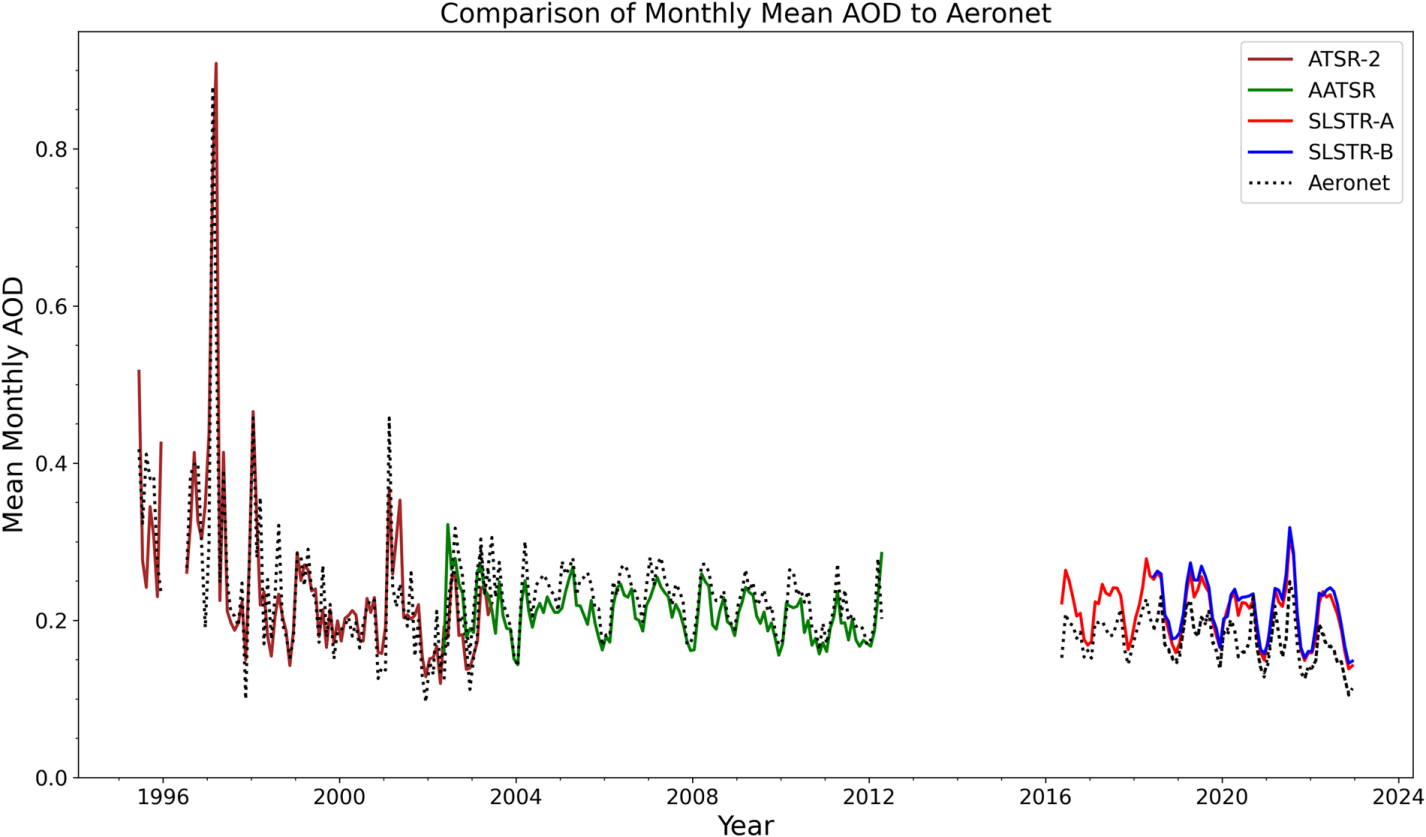
Timeline of monthly mean AOD_550_ from all four sensors with corresponding AERONET values for comparison. Points represent the global average, each month, of the daily mean values from AERONET stations or, of the daily mean values from the matched L3 instrument grid boxes in which they lie.

The full-length, global, L3C AOD values are compared to AERONET in Fig. 9. Daily average AOD data from AERONET stations were matched to data in the nearest daily L3C 1° × 1° satellite grid-box. A monthly average was computed for station locations with more than 3 successful matches in a given month using only those days with matched data. A global average was then calculated from these monthly averages for all locations. The timeline shows the satellite global mean AOD remains approximately constant over the period 2000–2023, intra-annual variability aside, whereas the AERONET values suggest a slight decline in the latter half of the period. This is consistent with the values in Tables 8, 9. As with the L2 comparisons, the over-representation of the Northern hemisphere in the AERONET station distribution will have an effect on global comparisons.

Comprehensive intercomparisons of different algorithm versions for (A)ATSR (v4.32, v4.33 and v4.35) and SLSTR (v1.12 and v1.14), along with comparisons to other instruments, are presented in the Aerosol CCI + Product Validation and Intercomparison Report^36^. Further intercomparisons between major satellite datasets and global model reanalyses for earlier AATSR versions have been published^37–39^ as well as an intercomparison of satellite datasets up to 2019 including SLSTR^40^. Below, we conduct intercomparisons to other satellite datasets over the coincident periods. The other (A)ATSR/SLSTR algorithms are the ATSR dual-view and single-view algorithms (ADV/ASV), the SLSTR dual-view and single-view algorithms (SDV/SSV) and the Oxford-RAL aerosol and cloud algorithm (ORAC), which have all been made available through the Copernicus Climate Data Store^11^. Further instruments are the Moderate Resolution Imaging Spectroradiometer (MODIS) on the Terra platform, the Multi-angle Imaging SpectroRadiometer (MISR), the Medium Resolution Imaging Spectrometer (MERIS) and the Ocean and Land Colour Instrument (OLCI). The properties of these instruments and datasets are summarised in Table 11.

**Table 11:** Summary of satellite aerosol datasets and properties referred to in the performance comparison.

Instrument	Date range	Algorithm version	Products	Frequency	Resolution L2 L3	Reference
ATSR-2	1995/06–2003/06	SU ATSR-2 v4.35 ADV/ASV v2.31	AOD, FMF	4-6 days	9 km, 1°	^10,50^
AATSR	2002/05–2012/04	SU ATSR-2 v4.35 ADV/ASV v2.31 ORAC v4.01	AOD, FMF	4 days	9 km, 1°	^10,50,51,52^
SLSTR-A	2016/05–	SU SLSTR v 1.14 SDV/SSV v2.30 ORAC v4.01	AOD, FMF	1-2 days	4.5 km, 1°	^11,53,51,52^
SLSTR-B	2018/05–	SU SLSTR v 1.14	AOD, FMF	1-2 days	4.5 km, 1°	^11^
MODIS Terra	2000/03 –	DT&DB, Collection 6.1	AOD, FMF (Ocean)	1-2 days	10 km, 1°	^54,55^
MISR	2000/03 –	MISR Standard Product, V23	AOD, FMF	6-7 days	4.4 km, 0.5°	^56,57^
MERIS	2002/05–2012/04	MERIS_XBAER v2.3	AOD	3-4 days	10 km, 1°	^58^
OLCI	05/2015 –	OLCI_XBAER v1.0	AOD	1-2 days	10 km, 1°	^59^

**Fig. 10:**
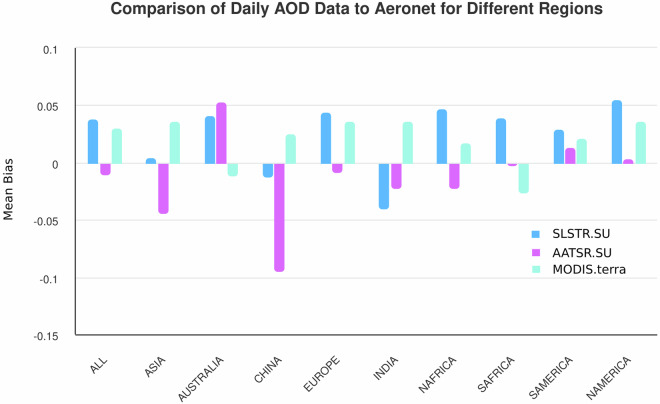
Comparison of L3 daily AOD values to AERONET for all AATSR, SLSTR-A and MODIS measurements between 2002 and 2022.

Figure 10 shows the mean bias with respect to AERONET values of L3C daily data from AATSR, SLSTR-A and MODIS in different regions. Each dataset has been individually matched to AERONET and as such are not co-incident with each other. The global biases for SLSTR-A and MODIS calculated in this way are similar, while the AATSR global bias has a very small magnitude. The AATSR bias is also low for most regions separately but does show a notable negative bias over China. Each dataset meets the GCOS requirement in different regions. While the size and magnitude of the bias of each instrument varies region by region, there are no readily-apparent, overall, systematic differences in their behaviour. In particular, the AATSR and SLSTR-A retrievals do not show any common patterns of exceptionally high- or low-bias behaviour in the same region which might have indicated a common deficiency in the representation of the aerosol types in those locations. Variations in aerosol composition have been incorporated in the retrieval algorithm by the of use the input aerosol type climatology for the mixtures within the fine and within the coarse mode. This is necessarily an approximation to reality in that it amounts to a typical composition for the month and location. As such, it is less representative when atmospheric aerosols arise from transient events or with high spatial variability.

It is readily apparent that the overall mean bias in Fig. 10 is not simply the sum over the individual regions. Rather, the overall mean bias is equivalent to the sum over every region weighted by the number of AERONET matches found in the region. As such, the overall mean bias, is strongly dependent on the geographical distribution of the stations. This distribution is weighted towards the Northern Hemisphere, to developed countries and to more easily accessible locations. It has also evolved over time. As a result, there are selection effects present in the mean bias statistics here and in Tables 8, 10 relative to actual global performance. Additionally, the distribution results in preferentially over-sampling what is the weaker, back-scatter direction for SLSTR with the potential to increase the mean bias relative to AERONET compared to (A)ATSR. It is also not clear to what extent the increase in the mean bias of the instruments is a result of the distribution of stations having changed over time. For similar reasons, in regions with relatively few stations, such as China, there is greater uncertainty in the mean bias values.

**Fig. 11:**
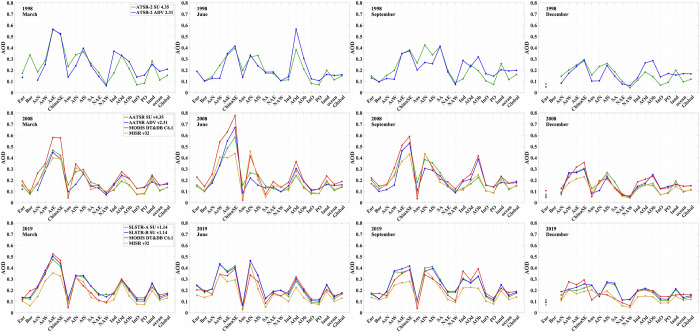
Comparison of the median retrieved AOD in the regions shown in Fig. 12 for each instrument to that from other sources. Top row: ATSR-2 AOD compared to the results from the “AATSR dual-view” (ADV) retrieval scheme^50^. Middle row: AATSR AOD compared to ADV retrievals and to AOD data derived from the Moderate Resolution Imaging Spectroradiometer (MODIS) and from the Multi-angle imaging spectroradiometer (MISR); both on the Terra platform; Bottom row: SLSTR AOD compared to MODIS and MISR data.

**Fig. 12:**
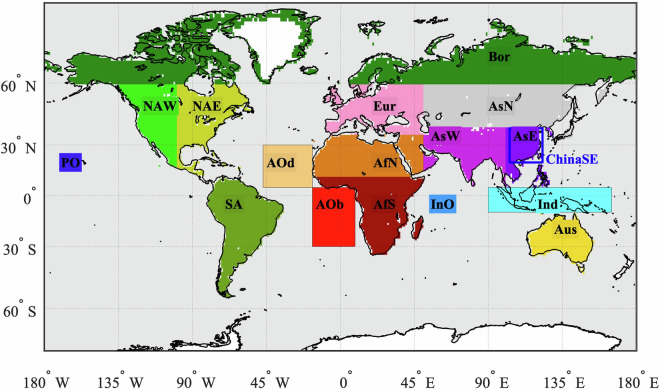
Regions used in the comparison of different datasets shown in Fig. 11.

AOD for selected months is compared to a different retrieval scheme and to other instruments in Fig. 11 for the regions shown in Fig. 12. The data points represent the area median AOD value from the L3C monthly products in each case. As such, they do not show the bias between the different datasets directly, since the spatial and temporal coverage varies, rather the comparison is between climatological values. The results for SLSTR-A and -B give an indication of how differences in temporal coverage can be reflected in monthly data. Similarly, the ADV retrievals for (A)ATSR give an indication of how different retrieval approaches for the same instrument can be reflected in monthly data.

When comparing SLSTR AOD to results from MODIS and MISR, there is no consistent pattern of any instrument always being higher or lower than the others. MISR, however, does tend to return the lowest AOD for most regions. For ocean and Asian land locations, SLSTR AODs generally lie close to the MODIS values, whereas MODIS results are closer to MISR for African and North American regions.

**Fig. 13:**
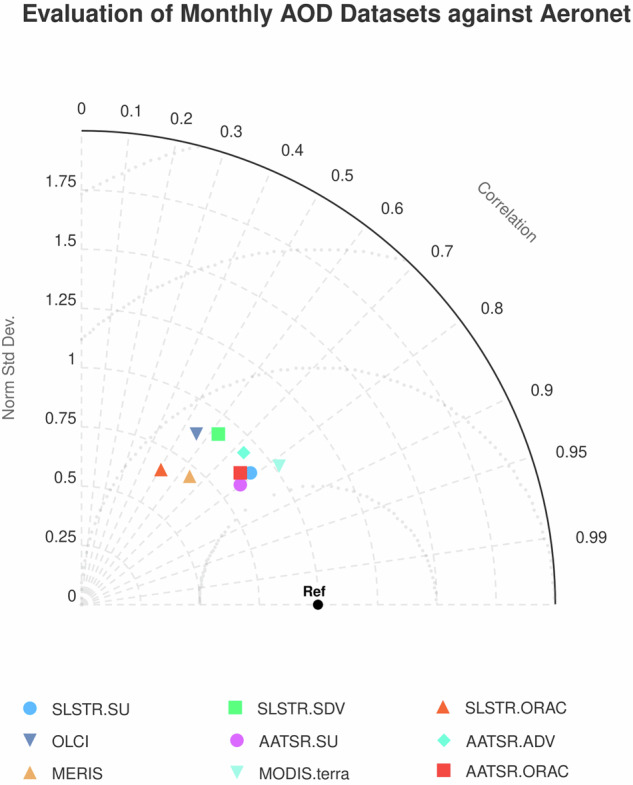
Taylor diagram comparing the performance of several L3 monthly AOD datasets using AERONET data as a reference. Points are plotted for AATSR and SLSTR-A from the datasets presented here as well as for those generated using the ORAC and ADV/SDV algorithms. Monthly AOD derived from MERIS, OLCI and MODIS (on the Terra platform) are also shown.

Several L3C monthly datasets are compared to the reference AERONET data in the form of a Taylor diagram^41^ in Fig. 13. The AATSR and SLSTR-A data presented here and the AATSR data generated with the ORAC algorithm have very similar performances when measured in this way. MODIS data has a comparable overall correlation with AERONET and also has a normalised standard deviation close to unity. It should be noted, however, that it is not clear that a value of unity is ideal for this statistic. The greater spatial but lower temporal sampling present in the satellite measurements contributing to a grid box stand in contrast to many AERONET point measurements at a fixed location. This may result in a systematic change in the variability of the mean AOD for grid boxes between satellite and AERONET datasets.

## Usage Notes

The stability of the predecessor versions (v4.33) of the combined ATSR-2/AATSR record has been assessed in the Copernicus Climate Change Service’s Product Quality Assessment Report^42^ and for AOD_550_ was found to be better than 0.01 per decade. Recent so far unpublished assessments with the Aeroval tool confirm for the full ATSR-2/AATSR (v4.35)/SLSTR (v1.14) record 1996–2022 an AOD_550_ stability better than 0.004 per decade^43^. This confirms the suitability of the data record for trend analysis. However, a user should be aware of the following remaining inconsistency (as shown in Table 8) in the combined data record of AOD_550_: the part of the record covering the two SLSTR instruments (2016–2022) has a higher bias (~0.05 on global average) than for the two ATSR instruments (1995–2012; ~0.02 on global average); these biases are larger for some regions and may add uncertainty to regional trend analyses. This dataset, based on the dual-view instrument series from ATSR-2 to SLSTR, is unique with regard to its historic length dating back to 1995, whereas MODIS and MISR started in 2000 and MERIS in 2002. In comparison to the other two dual-view records, this dataset provides the greatest coverage (especially for SLSTR) with overall similar quality (better than ORAC and comparable to ADV/SDV).

All the dataset files are produced in NetCDF-4 format with data arrays (called variables) containing the retrieved and additionally derived quantities listed in Table 4. FMF and AOD_550_ are the two directly retrieved quantities. As such, these are the two preferred variables for intercomparisons with other datasets. Other quantities have been included that are derived consistently with these two for user convenience. The uncertainties for AOD values contributing to the aggregation over the spatio-temporal scales included here cannot be assumed to be fully independent. The details of the scales and characteristics for correlations between contributing values is not fully understood. Reasonable assumptions have been made in propagating L2 uncertainties forward to L3 for AOD but statistical information on the contributing uncertainties is also included for further consideration if required. Care should be taken in particular when using uncertainties attributed to AOD at several different wavelengths. These AODs have all been derived to be self-consistent with AOD at 550 nm but are dependent on it.

It is intended that these datasets will be regularly extended it time and made available by EOCIS. The previous versions of the datasets (4.33 and 1.12) are currently accessible through the Copernicus Climate Change Service Climate Data Store and these will also be updated to versions 4.35.1 and 1.14.1 in the future.

## Data Availability

There are many tools for accessing and manipulating NetCDF data files^44–46^ as used in these datasets. Visualisation may be achieved simply with Panoply^47^. Sample Python code that accesses the datafiles and produced Fig. 3 is available^48^; based on an example from the Sea Surface Temperature CCI^49^.
